# Effective treatment of hypertension by recombinant *Lactobacillus plantarum* expressing angiotensin converting enzyme inhibitory peptide

**DOI:** 10.1186/s12934-015-0394-2

**Published:** 2015-12-21

**Authors:** Guilian Yang, Yanlong Jiang, Wentao Yang, Fang Du, Yunbao Yao, Chunwei Shi, Chunfeng Wang

**Affiliations:** College of Animal Science and Technology, Jilin Provincial Engineering Research Center of Animal Probiotics, Jilin Agricultural University, Changchun, 130118 China

**Keywords:** Hypertension, ACEIP, *Lactobacillus plantarum*, Antihypertensive

## Abstract

**Background:**

Hypertension is considered the most serious risk factor for cardiovascular disease. Angiotensin-converting enzyme inhibitory peptides (ACEIPs), which are made from tuna frame protein (TFP) and yellow fin sole frame protein (YFP), have been used previously to treat hypertension. However, the production of these short peptides is usually dependent on enzymatic hydrolysis, resulting in a digested mixture that makes it difficult to purify the ACEIPs. Although it has been reported that ACEIPs could be produced in recombinant *Escherichia coli* strains, the use of *lactic acid bacteria* in the production of ACEIPs has not been demonstrated.

**Results:**

In this study, the ACEIP coding sequences from TFP and YFP were joined through an arginine linker and expressed in the *Lactobacillus plantarum* (*Lb. plantarum*) NC8 strain by an inducible vector pSIP-409. Then, the antihypertensive effects were determined in the model of spontaneously hypertensive rats (SHRs) by measuring the blood pressure, hematology, blood biochemistry and nitric oxide (NO), endothelin (ET) and angiotensin II (Ang II) levels. The results showed that oral administration of recombinant *Lb. plantarum* NC8 (RLP) significantly decreased systolic blood pressure (*P* < 0.01) during treatment, which lasted for at least 10 days after the last dose. Furthermore, the presence of RLP resulted in an increased level of NO, as well as decreased levels of ET and Ang II in plasma, heart, and kidney. In addition, a dramatically decreased triglyceride level was also observed even though there was no significant change in hematology or blood biochemistry. Although some drawbacks were still observed, such as the presence of an antibiotic selection marker, no obvious side effects or bacterial translocation were observed in vivo, indicating the potential application of RLP in the treatment of hypertension.

**Conclusion:**

These results demonstrated the effectiveness and safety of RLP on the treatment of hypertension.

## Background

Approximately twenty percent of the world’s population is susceptible to serious hypertension (HTN), which is a frequent cause of cardiovascular diseases, the leading cause of death globally [1]. As a frequent and chronic age-related disorder, it has been demonstrated that HTN is usually accompanied with other cardiovascular risk factors, including abdominal obesity, dyslipidemia, glucose intolerance, hyperinsulinemia, and hyperuricemia [2]. According to previous reports, the kidneys play a central part in the pathophysiology of essential hypertension; therefore, kidney disorders, such as renal ischemia [3], have been considered the most critical risk factor for HTN. In addition, obstructive sleep apnea has been newly recognized as a secondary cause of HTN according to the Seventh Joint National Committee [4]. Other factors such as hypothyroidism [5] and nitric oxide deficiency [6] have also been identified as risk factors for HTN.

The renin-angiotensin system (RAS) plays an important role in maintaining blood pressure homeostasis in addition to the fluid and salt balance in mammals [7]. Angiotensin-converting enzyme (ACE) is a dipeptidyl carboxypeptidase that plays an important physiological role in regulating blood pressure by virtue of the RAS [8]. In the RAS, ACE converts inactive peptide angiotensin I (Ang I) into powerful vasoconstrictor angiotensin II (Ang II) and inactivates the catalytic function of bradykinin [9–11].

Since the discovery of ACE inhibitors in snake venom [12], many synthetic inhibitors have been developed and are currently used in the treatment of essential hypertension and heart failure in humans; these inhibitors include captopril [13], enalapril [14], alacepril [15] and lisinopril [16]. However, these synthetic ACE inhibitors are believed to have certain negative effects such as cough, taste disturbances, skin rashes and other serious diseases [17]. Recently, a series of natural ACE inhibitory peptides (ACEIPs) have been produced either by protein hydrolysis [18] or recombinant technologies [19, 20] without any obvious side effects, indicating their great application potential. One of these ACEIPs belongs to the family of the yellowfin sole (*Limanda aspera*) frame protein (YFP), with a molecular mass of 1.3 kDa and 11 amino acids. After peptide ingestion, blood pressure significantly decreased in spontaneously hypertensive rats (SHRs) [21]. Similar beneficial effects were also observed during a study in which a 21-amino-acid peptide from tuna frame protein (TFP) dramatically decreased blood pressure in the SHR model [22].

*Lactic acid bacteria* (LAB) include a group of Gram-positive, nonsporulating cocci and rod-shaped anaerobic bacteria, producing lactic acid as the major metabolite of carbohydrate fermentation. LAB have been used widely in the production of fermented foods for centuries, and several LAB have been recognized as probiotics because of their wide health-promoting effects in humans. The most widely documented effects of LAB include improved immune function [23], prevention and reduced intensity of diarrhea [24], and reduced lactose intolerance [25]. One of the LAB that has been studied in the most detail is *Lactobacillus plantarum* (*Lb. plantarum*). In addition to its natural beneficial properties, *Lb. plantarum* has been genetically engineered to express protective antigens [26, 27], β-galactosidase [28], or oxalate decarboxylase [29] for medical applications. Most recently, the engineered *Lb. plantarum* has also been considered an alternative strategy for delivering DNA vaccines [30].

In the present study, we investigated the production of recombinant ACEIPs from both YFP and TFP in the *Lb. plantarum* NC8 strain and evaluated the biological and safety effects. The results show that oral administration of RLP dramatically decreases blood pressure, endothelin (ET) and Ang II production, and triglyceride levels with no observed side effects, indicating its potential application in hypertension and related diseases.

## Results

### Construction of recombinant pSIP409-ACEIP vector and expression of recombinant ACEIP in *Lb. plantarum* NC8

**Fig. 1:**
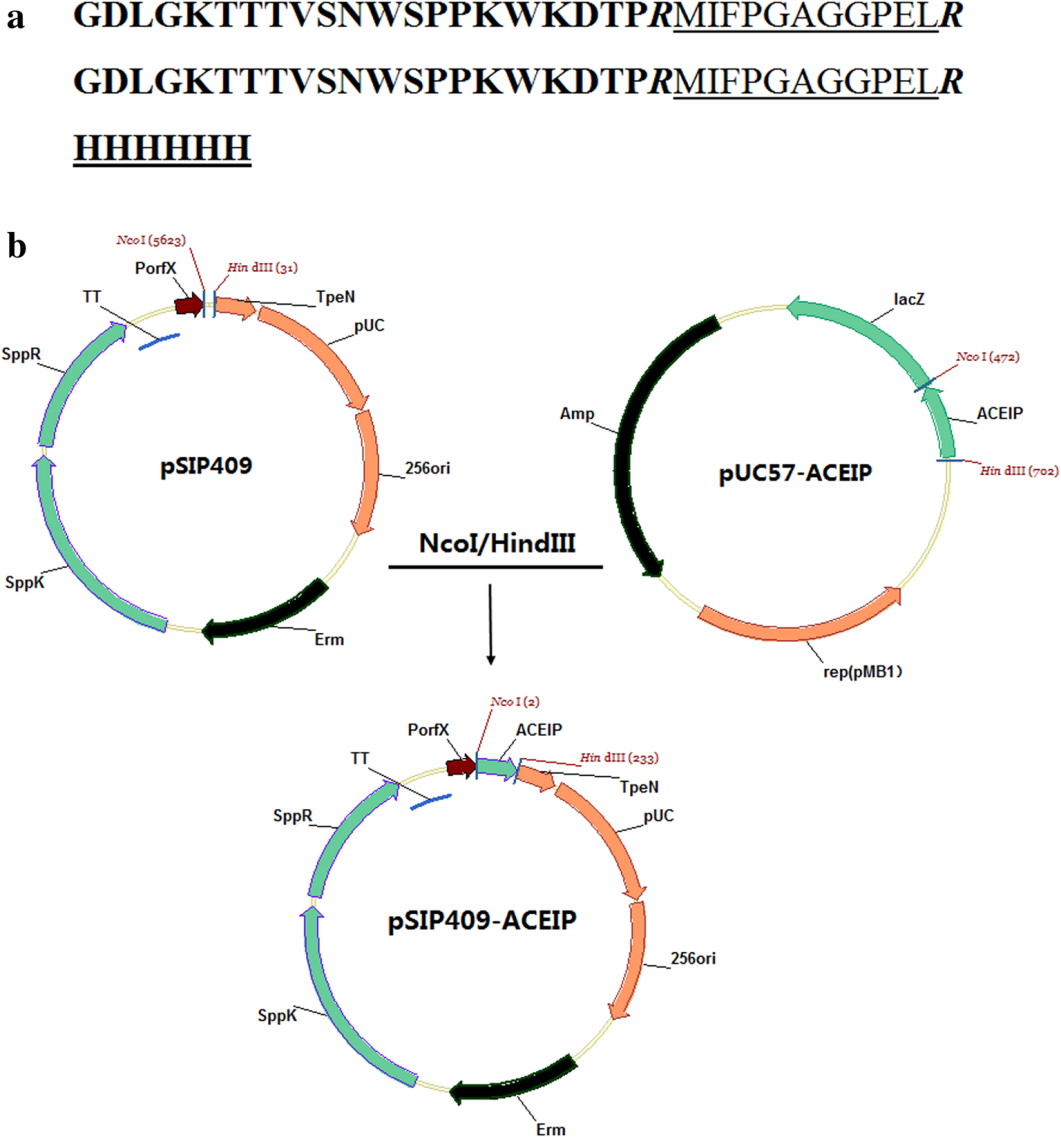
Construction of recombinant plasmid pSIP409-ACEIP. **a** Designed peptide sequences according to the synthesized oligonucleotides. *Black bold*, peptides of tuna frame protein; *Black bold italic*, arginine linker; *Underlined*, peptides of limanda aspera frame protein; *Black bold underlined*, His tag. **b** Illustration of pSIP409-ACEIP. Encoding sequences of angiotensin-converting enzyme inhibitory peptide (ACEIP) were digested by *Nco*I and *Hin*dIII and were ligated with pSIP409 that was digested with the same enzymes. *256rep* replication origin for *Lactobacillus*, *ermL* erythromycin-resistance marker, *P*_*sppIP*_ and *P*_*orfX*_ inducible promoters, *sppK* and *sppR* histidine protein kinase and response regulator, respectively

**Fig. 2:**
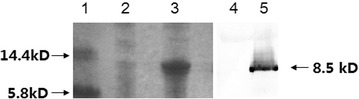
Protein profiles of *Lb. plantarum* NC8 on SDS-PAGE. *Lb. plantarum* NC8 harboring pSIP409-ACEIP was induced with 50 ng/mL of sakasin-P (SppIP)-inducing peptide at OD_600_ = 0.6 and then the induced cells were harvested at 7 h by centrifugation and were suspended in 50 mM phosphate buffer, followed by sonic disruption. The cell-free extract was analyzed on 17.5 % sodium dodecyl sulfate–polyacrylamide gel electrophoresis (SDS-PAGE) and subjected to western blotting (WB) using rabbit anti-His polyclonal antibody as prime antibody. *Lane 1* protein marker; *lane 2* non-induced *Lb. plantarum* NC8 (SDS-PAGE); *lane 3* induced *Lb. plantarum* NC8 at 7 h (SDS-PAGE); *lane 4* non-induced *Lb. plantarum* NC8 (WB); *lane 5* induced *Lb. plantarum* NC8 at 7 h (WB)

The encoding sequences of peptides from YFP and TFP were synthesized and joined through an arginine linker as shown in Fig. 1a. After digestion by *Nco*I and *Hin*dIII, the gusA gene present in pSIP409 was replaced with genes encoding an ACEIP fusion protein, which was confirmed by restriction endonuclease digestion and sequencing (data not shown), yielding the recombinant expression vector pSIP409-ACEIP (Fig. 1b). The SDS-PAGE and western blotting results showed that a specific protein band of approximately 8.5 kDa was expressed in *Lb. plantarum* NC8 (Fig. 2).

### Antihypertensive activity of recombinant *Lb. plantarum* NC8 (RLP)

**Fig. 3:**
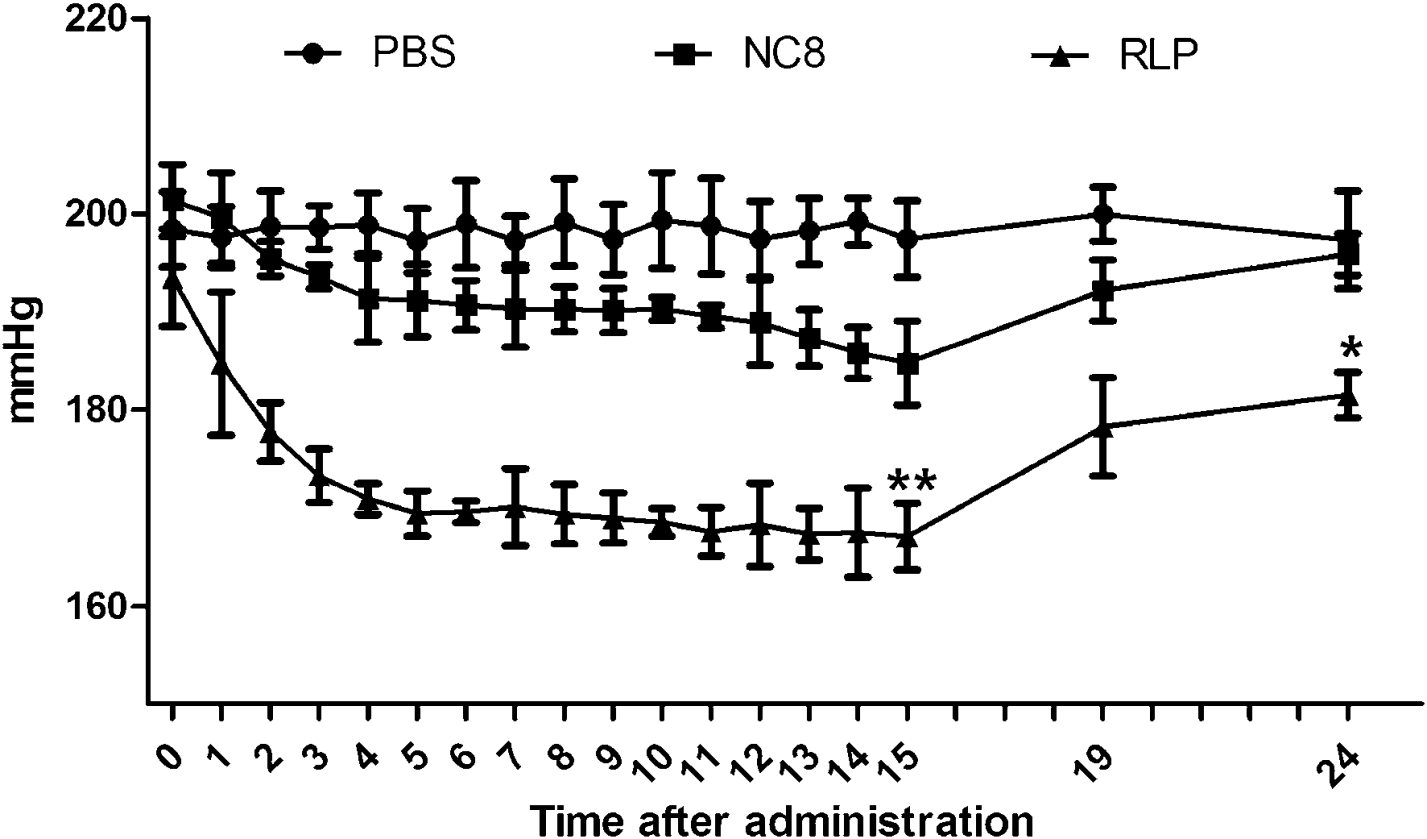
Change of systolic blood pressure (SBP) after oral administration of recombinant *Lb. plantarum* NC8 (RLP) strain in spontaneously hypertensive rat. The rats were treated orally with either RLP or *Lb. plantarum* NC8 (NC8) at a dose of 2 × 10^11^ CFU for 14 continuous days, whereas additional PBS-treated rats were included as controls. The SBP was continuously determined during the first 15 days and then at day 19 and day 24 as described in the methods section. The statistical significance was calculated by one-way ANOVA test. *P < 0.05; **P < 0.01 vs. PBS control

Antihypertensive activity of RLP was evaluated by measuring the systolic blood pressure (SBP) every day during the first 15 days and then on day 19 and day 24 (Fig. 3). The results showed that the SBP in the RLP-treated group decreased dramatically as time elapsed, with the lowest value of 167.111 ± 3.418 mmHg occurring on the 15th day, which was significantly lower (*P* < 0.01) than the 184.810 ± 4.305 mmHg in the *Lb. plantarum* group and the 197.443 ± 3.893 mmHg in the PBS group. Although the SBP values in the RLP group increased after the last dose at day 15, the antihypertensive function of RLP was maintained for at least 10 days because the SBP of the RLP-treated rats (181.517 ± 2.312 mmHg) was significantly lower than that of the *Lb. plantarum* treated rats (195.876 ± 2.109 mmHg) and the PBS control rats (197.376 ± 4.982 mmHg) on the 24th day (*P* < 0.05). Interestingly, the PBS control rats maintained hypertensive status during the entire study. In addition, we observed that the administration of *Lb. plantarum* also decreased the SBP level in rats compared with the PBS controls, with lowest values of approximately 185 mmHg on day 15. All the results mentioned above clearly demonstrated that the administration of RLP in rats significantly decreased the SBP level because of the presence of recombinant ACEIP.

### Hematology and blood biochemistry

The hematological parameters and blood biochemical indices in each group were also determined (Table 1). The values of hematocrit (HCT), total plasma cholesterol (TCHE) and triglyceride (TG) were significantly decreased in the RLP-treated rats compared with the PBS controls, whereas the levels of TCHE and TG in the RLP-treated rats were even lower than in the rats treated with NC8 alone (*P* < 0.05).

**Table 1:** Haematology and blood biochemistry measurements (mean ± SEM) of spontaneously hypertensive rats (SHR)

Parameters	PBS	NC8	RLP
WBC (10^9^/L)	5.133 ± 1.935	5.567 ± 0.929	5.400 ± 2.272
RBC (10^12^/L)	6.810 ± 0.764	6.727 ± 0.748	7.620 ± 0.735
HGB (g/L)	94.33 ± 14.154	102.33 ± 6.658	113.00 ± 10.583
HCT (%)	0.308 ± 0.013	0.320 ± 0.047	0.382 ± 0.034*
MCV (fL)	50.500 ± 0.700	50.000 ± 1.200	50.133 ± 0.351
MCH (pg)	14.133 ± 0.902	15.667 ± 0.757	14.833 ± 0.058
MCHC (g/L)	296.00 ± 30.610	314.00 ± 22.113	295.67 ± 1.155
PLT (10^9^/L)	533.33 ± 59.181	589.33 ± 36.611	661.67 ± 289.096
W-SCR (%)	0.752 ± 0.064	0.607 ± 0.132	0.793 ± 0.159
W-SCC (%)	3.000 ± 0.600	3.133 ± 0.231	4.400 ± 2.193
RDW-SD (fL)	29.000 ± 0.100	29.033 ± 0.379	28.800 ± 0.625
RDW-CV (%)	0.147 ± 0.001	3.147 ± 5.198	0.148 ± 0.009
PDW (fL)	7.500 ± 0.100	7.733 ± 0.451	7.967 ± 0.643
MPV (fL)	6.200 ± 0.100	6.433 ± 0.231	6.700 ± 0.361
P-LCR (%)	0.030 ± 0.011	0.042 ± 0.009	0.047 ± 0.013
UREA (mmol/L)	8.343 ± 1.517	8.340 ± 0.963	7.700 ± 1.908
GLUC (mmol/L)	16.570 ± 2.370	14.300 ± 2.383	15.480 ± 1.019
TCHE (mmol/L)	1.360 ± 0.044	1.333 ± 0.125	1.100 ± 0.050*^,#^
TG (mmol/L)	1.247 ± 0.025	1.213 ± 0.176	0.750 ± 0.181*^,#^

The rats were divided into three groups and subjected to either PBS, *L. planturm* NC8 (NC8) or recombinant *Lb.Plantarum* NC8 (RLP) treatment for continuous 14 days, and then 5 rats from each group were selected at random at day 14 and the cardiac puncture samples were collected and the following values were measured

*WBC* white blood cell, *RBC* red blood cell, *HGB* haemoglobin, *HCT* hematocrit, *MCV* mean corpuscular volume, *MCH* mean corpuscular haemoglobin, *MCHC* mean corpuscular haemoglobin concentration, *PLT* platelet counts, *W-SCR* lymphocyte percentage, *W-SCC* absolute value of lymphocyte, *PDW* platelet distribution width, *MPV* mean platelet volume, *P-LCR* platelet large cell ratio, *UREA* urea nitrogen, *GLUC* total plasma glucose, *TCHE* total plasma cholesterol, *TG* triglyceride

The statistical significance was calculated using one way ANOVA.* *P* < 0.05 vs. PBS control. ^#^ *P* < 0.05 vs. NC8 control

### Effects of RLP on nitric oxide (NO), ET and Ang II levels in plasma, kidney and heart

**Fig. 4:**
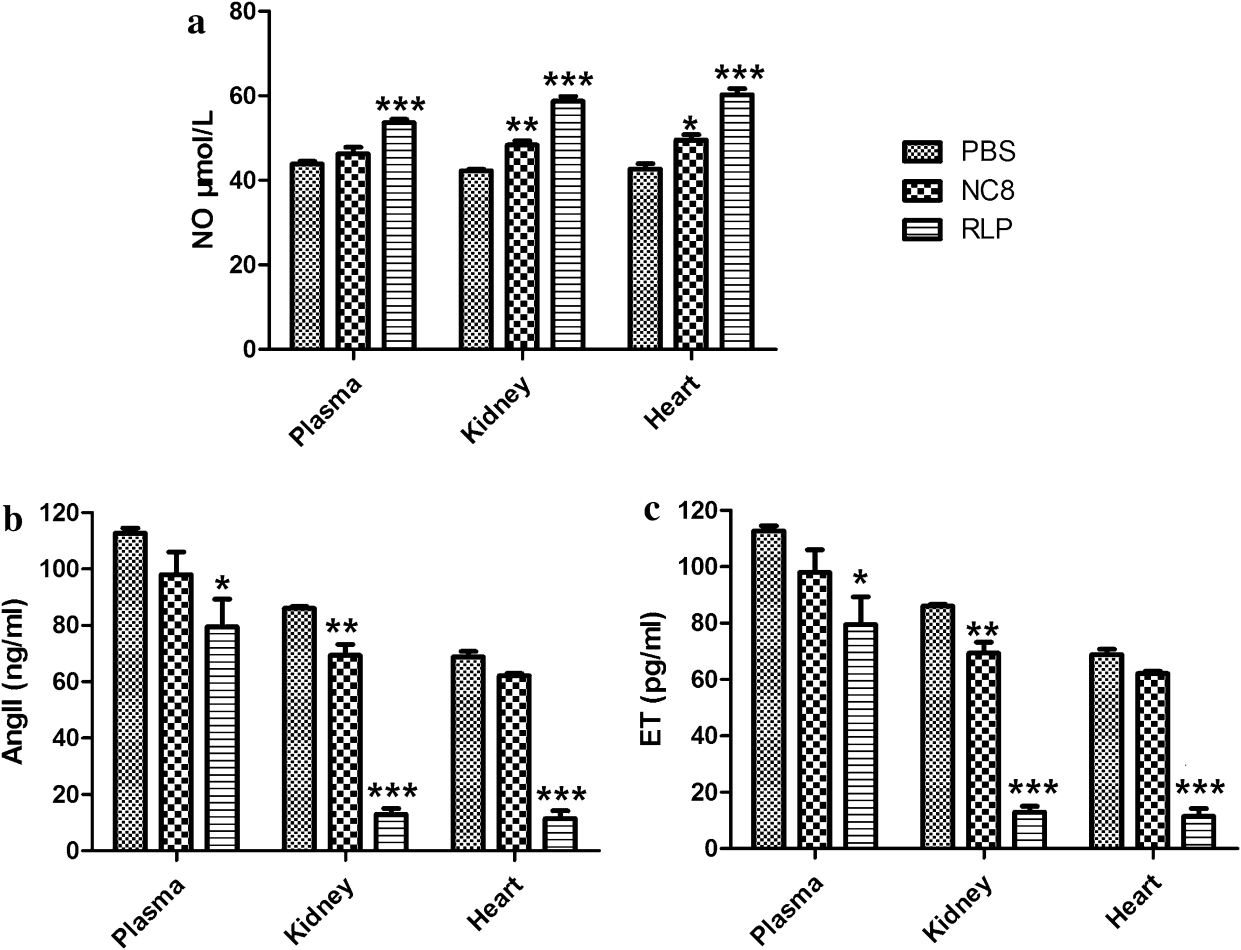
Effects of recombinant *Lb. plantarum* NC8 (RLP) on the levels of nitric oxide (NO) (**a**), angiotensin II (Ang II) (**b**) and endothelin (ET) (**c**) in the plasma, kidneys and hearts of spontaneously hypertensive rats. The rats were orally treated with either RLP, *Lb. plantarum* NC8 or PBS for 14 continuous days, and then the samples were collected from 5 rats in each group at day 14. The measurements of NO, ET and Ang II levels were performed using ELISA kits, and the statistical significance was calculated by one-way ANOVA test. *P < 0.05; **P < 0.01; ***P < 0.001 vs. PBS control

The NO values in plasma, kidneys and hearts of the RLP-treated rats increased significantly compared with those in the PBS control rats (*P* < 0.001) (Fig. 4a). By contrast, the Ang II (Fig. 4b) and ET (Fig. 4c) values in plasma, kidneys and hearts of the RLP-treated rats decreased dramatically compared with those of the PBS controls. In addition, we also noticed that the presence of NC8 alone also increased the levels of NO and decreased the levels of AngII and ET compared to the PBS controls (Fig. 4).

### Safety evaluation of RLP in mice

To determine the safety of RLP, an acute toxicity test (ATT), a bone marrow cell micronucleus test (BMCMT) and a bacterial dissemination test (BDT) were performed. The results of ATT did not show any potential adverse effect on general health status, body weight gain, morphology (data not shown) or weight of spleen and liver (Table 2). The results of the BMCMT test demonstrated that the micronucleus ratio in RLP-treated mice (2.7 ‰) was almost the same as that of the negative PBS control (2.5 ‰), whereas the ratio in cyclophosphamide (CP)-injected mice was significantly higher (27.3 ‰). In addition, no viable bacteria were recovered from blood, spleen or liver in any groups according to the results of the BDT (data not shown), indicating that repeated administration of a high dose of RLP did not induce bacteria translocation. Consistent with a previous study, body weight gain was still not affected by RLP treatment (data not shown). All the results mentioned above demonstrated that oral administration of RLP was safe, at least in experimental conditions. However, there was still an obvious limitation in our system: the presence of an antibiotic selection marker (Erm^+^) would prevent us from using this system in a clinical trial. Thus, further studies to exchange the Erm-resistant gene with a food-grade selection marker would be necessary.

**Table 2:** The change of spleen, liver and body weight ratio

Groups	LW/BW	SW/BW
PBS^a^	0.050 ± 0.0082	0.0045 ± 0.0016
4.5 × 10^7^CFU^a^	0.055 ± 0.0058	0.0047 ± 0.0013
4.5 × 10^9^CFU^a^	0.046 ± 0.0079	0.0042 ± 0.0017
PBS^b^	0.054 ± 0.0057	0.0059 ± 0.0014
4.5 × 10^7^CFU^b^	0.065 ± 0.0063	0.0052 ± 0.0018
4.5 × 10^9^CFU^b^	0.045 ± 0.0061	0.0051 ± 0.0017

The liver and body weight ratio (LW/BW), spleen and body weight ratio (SW/BW) were calculated in two separate studies, including the acute toxicity test (ATT, labeled as “a”) and bacterial dissemination test (BDT, labeled as “b”). There were three groups in both studies, PBS control, low dose (4.5 × 10^7^ CFU) and high dose (4.5 × 10^9^ CFU) treatment. The mice in ATT study were orally administrated for 3 days and observed for additional 7 days, whereas the mice in BDT assay were treated for 4 weeks

## Discussion

Oral administration of ACEIPs has been demonstrated to be effective in the control of HBP in SHR models even though the antihypertensive effect could last for only a few hours after treatment cessation [31–33]. One of the possible explanations for the short-lasting effects was that the administered ACEIP could be digested rapidly in vivo because of the direct exposure to the host’s internal environment. Accordingly, it was reasonable to deduce that the ACEIP delivered by a bacteria host would perform better during the treatment of HBP because of the protective effects provided by the host strains. Our results demonstrated that the antihypertensive effect of RLP-expressing ACEIP lasted at least an additional 10 days after the last administered dose.

In fact, the administration of *Lb. plantarum* alone also decreased the BP levels during at least the first 15 days, with the lowest value at day 9 (Fig. 3). It has previously been reported that the administration of probiotic-fermented products had some beneficial effects in reducing blood pressure in patients with hypertension [34–36], by releasing bioactive peptides such as ACEIP during the fermentation process [37]. Another possible explanation of the observed effect involve the maintenance of the gut microbiota balance, which has gained increasing attention recently in the field of human health. The imbalance of gut microbiota has been primarily associated with gastrointestinal health disorders and other diseases such as Parkinson’s disease [38], fatty liver disease [39], diabetes [40] and hypertension [41]. Therefore, the administration of probiotics could possibly restore the microbiota balance, thus improving endothelial dysfunction, vascular inflammation, vascular oxidative stress, cardiac and renal hypertrophy to reduce high BP in the SHR model [37].

In the present study, the mechanism of RLP in lowering blood pressure was further identified. The ET and Ang II levels in plasma, kidneys and hearts of the RLP-treated SHRs dramatically decreased compared with that of the rats treated with *Lb. plantarum* NC8 or PBS. However, the NO values in the RLP-treated SHR group increased significantly compared with that of the two control groups. NO is mainly produced by vascular endothelial cells and is considered a strong vasodilating factor [42]. The presence of other types of ACEIPs has been demonstrated to be effective in reducing the NO level in serum and kidneys [43, 44]. In our case, RLP expressing the ACEIP fusion protein may promote the activity of NO synthesis and increase the release of NO, which could be responsible for the observed lower SBP in rats. Currently, it is believed that ET belongs to a family of vasoconstrictive peptides and is distributed in the cardiovascular system. Previous studies have identified that Ang II could stimulate endothelial cells to produce ET [45]. In this study, the presence of RLP repressed the expression of Ang II and then resulted in the observed lower ET compared with that of the control rats, which could be responsible for the decreased SBP in the SHRs. However, the detailed mechanism is still not clear; therefore, further investigation will be necessary. In addition, this study also demonstrated that the oral administration of RLP could dramatically decrease blood TCHE and TG levels, consistent with the findings of previous reports with regard to the effects of ACEIP [43, 44]. The safety of RLP was also examined because probiotics should be innocuous whenever they are used; no obvious adverse effects or bacterial translocation were observed.

However, improvements to our system could be made as a result of this study. The first option is to explore a food-grade non-antibiotic selective marker, such as alanine racemases [46], to exchange the erythromycin-resistance gene. Another reasonable approach is to make use of surface anchoring [47] or secreting methods [48] to enhance the interaction of ACEIPs with the host environment, which have been shown to be better in inducing an immune response than the intracellular expression system that was used in our study.

## Conclusions

In this study, we constructed an effective RLP strain that expressed two sources of ACEIP by protein fusion. The oral administration of RLP significantly decreased the SBP, TG, ET and Ang II levels in the SHR model and increased the NO level. Most importantly, this is the first report showing that ACEIPs could be produced in LAB; oral administration of RLP leads to the longest decreased effects on SBP currently found in the literature.

## Methods

### Bacterial strains, plasmids and animals

The bacterial strains and plasmids used in this study are listed in Table 3. *E. coli* DH5α cells were grown in Luria–Bertani medium at 37 °C with shaking, and *Lb. plantarum* NC8 cells [49] were grown aerobically in MRS medium at 30 °C without shaking. Solid media were prepared by adding 1.5 % (w/v) agar to the broth. When required, erythromycin was added as follows: 200 μg/mL for *E. coli* and 50 μg/mL for *Lb. plantarum.* The *E. coli*-*Lactobacillus* shuttle vector pSIP409 [49] was used as the expression vector through the entire study. SHRs with a tail SBP greater than 180 mmHg and BALB/c mice were obtained from Beijing Vital River Laboratory Animal Technology Co. Ltd. (Beijing, China). SHRs were housed individually in steel cages in a room kept at 24 °C with a 12 h light–dark cycle and were fed a standard laboratory diet. Tap water was freely available.

**Table 3:** Bacterial strains and plasmids used in this study

Plasmids or strains	Description	Source
pUC57-ACEIP	pUC57 with ACEIP; Amp^r^; 2.71 kb	BGI Beijing Corporation, China
pSIP409	p256rep/pUC(pGEM) ori:P_orfX_-gusA: :Em^r^	Provided by Dr. Lars Axelsson, Senior Research Scientist, Norwegian Food Research Institute
pSIP409-ACEIP	p256rep/pUC(pGEM) ori:P_orfX_-ACEIP: :Em^r^	This study
*E.coli* DH5α	Host strain	TaKaRa Corporation, Japan
*L. plantarum* NC8	Host strain, Plasmid-free, silage isolate	Provided by Dr. Lars Axelsson, Senior Research Scientist, Norwegian Food Research Institute

### Design of ACEIP oligonucleotides

The ACEIP oligonucleotides were designed based on previously published peptide sequence information [21, 22]. In detail, the 21-mer peptide (Gly-Asp-Leu-Gly-Lys-Thr-Thr–Thr-Val-Ser-Asn-Trp-Ser-Pro-Pro-Lys-Try-Lys-Asp-Thr-Pro) from TFP and 11-mer peptide (Met-Ile-Phe-Pro-Gly-Ala-Gly–Gly-Pro-Glu-Leu) from YFP were connected by an arginine linker, followed by another duplicate repeat and a 6× His tag (Fig. 1a). The corresponding nucleotides were codon optimized to maximize the expression in both *E. coli* and *Lb. plantarum*. The complementary oligonucleotides containing two restriction sites (*Nco*I and *Hin*dIII) were synthesized by BGI Beijing Corporation (Beijing, China), namely pUC57-ACEIP.

### Construction of recombinant plasmid and transformation

The ACEIP-containing DNA fragment was released from pUC57-ACEIP by double digestion with *Nco*I and *Hin*dIII and was inserted into pSIP409 digested with the same enzymes, yielding pSIP409-ACEIP (Fig. 1b). *E. coli* DH5α was used as the recipient strain for the recombinant plasmids, and *Lb. plantarum* NC8 was selected as the expression host. Competent *Lb. plantarum* were transformed by electroporation as described previously [30] with some modifications. In detail, *Lb. plantarum* was grown overnight in MRS broth and then inoculated to an optical density of 0.3 at 600 nm in MRS with 2 % glycine and incubated at 30 °C. At an optical density of 0.6 at 600 nm, the cells were chilled on ice and harvested, washed twice with ice-cold wash buffer and resuspended in 1/100 culture volume of electrode buffer. The competent cells were then subjected to a 2.5 kV, 5 ms electric pulse in a 0.2 cm cuvette, using a Gene Pulser (Bio-Rad, Richmond, CA, USA). Immediately after the pulse, cells were transferred to the 800 μL of MRS broth and were incubated at 30 °C for 2 h. The cells were plated on MRS agar containing the required antibiotic and were incubated for 24 h at 30 °C until visible colonies were observed.

### Expression and identification of ACEIP protein in *Lb. plantarum* NC8

*Lb. plantarum* NC8 harboring pSIP409-ACEIP was induced with 50 ng/mL of sakasin-P (SppIP)-inducing peptide at OD_600_ = 0.6. A final concentration of 5 mM MnCl_2_ was added along with the peptide inducer. The induced cells were harvested at 7 h by centrifugation and were suspended in 50 mM phosphate buffer, followed by sonic disruption. The cell-free extract was analyzed on 17.5 % sodium dodecyl sulfate–polyacrylamide gel electrophoresis (SDS-PAGE) and subjected to western blotting assay using rabbit anti-His polyclonal antibody (Sigma) as prime antibody.

### Animal experiments

Twelve-week-old male SHRs (weight 200–260 g) were randomly divided into three groups, with 10 rats in each group. The rats in group I were orally treated with *Lb. plantarum* NC8 harboring pSIP409-ACEIP induced by 50 ng/mL SppIP for 7 h at a dose of 2 × 10^11^ CFU for 14 continuous days; the rats in group II and III were exposed to either an equal dose of *Lb. plantarum* NC8 or PBS, respectively, as negative controls. The SBP was continuously determined during the first 15 days and then at day 19 and day 24 using the tail-cuff method with a non-invasive blood pressure BP-98A measuring system (Softron BP-98A, Tokyo, Japan) as described previously [50]. Five rats from each group were euthanized on the 14th day, and samples were collected for analysis of hematology, blood biochemistry, NO, ET and AngII levels.

### Hematology and blood biochemistry analyses

Blood samples were obtained from a cardiac puncture using EDTA-treated tubes. Total red blood cell (RBC) and white blood cell (WBC), hemoglobin (HGB), hematocrit (HCT), mean corpuscular volume (MCV), mean corpuscular hemoglobin (MCH), mean corpuscular hemoglobin concentration (MCHC), platelet counts (PLT), lymphocyte percentage (W-SCR), absolute value of lymphocyte (W-SCC), red blood cell volume distribution width-standard deviation (RDW-SD), red blood cell volume distribution width-coefficient of variation (RDW-CV), platelet distribution width (PDW), mean platelet volume (MPV) and platelet large cell ratio (P-LCR) were determined using a KX-21 N hematology Analyzer (Sysmex, Tokyo, Japan). Following completion of the hematology assays, plasma was separated from blood samples. The levels of total plasma glucose (GLUC), urea nitrogen (UREA), total plasma cholesterol (TCHE) and triglyceride (TG) were determined on a BS-400 Automatic biochemical analyzer (Mindray, Shenzhen, China).

### Measurement of NO, EU and AngII levels

The 2 mL blood samples that were mentioned above were collected and centrifuged at 1000×*g* for 15 min at 4 °C to collect the plasma. In addition, the heart and kidney samples were collected after homogenization by centrifugation at 1000×*g* for 20 min at 4 °C. Plasma and tissue homogenates were separated and stored at −70 °C until use. The concentrations of NO, ET and Ang II were determined by enzyme-linked immunosorbent assay (ELISA) kits (Rapidbio Inc, USA).

### Safety evaluation of RLP

To evaluate the safety of RLP, ATT and BMCMT assessments were performed as described previously with some modifications [51, 52]. Specifically, 6–8 week old BALB/c mice were divided randomly into three groups with 10 mice per group; the mice were subjected to either 4.5 × 10^9^ CFU or 4.5 × 10^7^ CFU RLP resuspended in 0.1 mL PBS. An additional 0.1 mL PBS served as the negative control. The animals were continuously treated for 3 days and were observed for 7 days. Behavioral changes, including body weight, morbidity, mortality and clinical signs of toxicity, were recorded during experiments. On day 8, the mice were killed by decapitation under anesthesia, and the spleen and liver were collected to calculate the ratio of spleen/liver to body weight and to perform histological analysis. Additionally, the other organs were also evaluated to determine the presence of any pathological changes.

To perform the BMCMT assay, 30 BALB/c mice were randomly divided into three groups with 10 mice per group. The RLP group was orally treated with a dose of 4.5 × 10^8^ CFU in 0.1 mL PBS for 5 continuous days, whereas the negative control group was treated with 0.1 mL PBS for 5 days and the positive control group was intraperitoneally injected with CP 50 mg/kg (BW) on the 5th day. Twenty-four hours after the last dose, the mice were sacrificed by cervical dislocation, and bone marrow cells were collected immediately as described before [52]. One thousand polychromatic erythrocytes (PCE) were recorded per mouse, and the frequency of micronucleated cells was calculated by counting them and dividing by the total number of polychromatic erythrocytes.

### Determination of RLP dissemination

The possibility of bacterial translocation in immunized mice was also evaluated as described previously with some modifications [53]. A total of 30 6–8 week old BALB/c mice were randomly divided into three groups with 10 mice per group. The mice were daily treated with either 4.5 × 10^7^ CFU RLP (low dose) or 4.5 × 10^9^ CFU RLP (high dose) that was resuspended in 0.1 mL PBS for 4 continuous weeks, whereas the administration of 0.1 mL PBS was used as the negative control. Next, all the mice were sacrificed, and the spleen and liver of each individual mouse were aseptically removed, weighted and placed immediately into sterilized PBS at a ratio of 1 mL/g. The samples were then mechanically homogenized and 0.1 mL samples were used on erythromycin-selective MRS agar plates. The presence of antibiotic resistant colonies was then determined after a further 48 h culture at 37 °C.

### Statistical analysis

All data were expressed as the mean ± SEM. Statistical analysis was performed using a two-tailed Student’s t test with GraphPad Prism 5.0 (GraphPad Software).

## Abbreviations

ACE: angiotensin converting enzyme
ACEIP: angiotensin converting enzyme inhibitory peptides
RAS: renin-angiotensin system
LAB: *lactic acid* bacteria
SppIP: sakasin-P
SHR: spontaneously hypertensive rat
SBP: systolic blood pressure
RBC: red blood cell
WBC: white blood cell
HGB: haemoglobin
HCT: hematocrit
MCV: mean corpuscular volume
MCH: mean corpuscular haemoglobin
MCHC: mean corpuscular haemoglobin concentration
PLT: platelet counts
W-SCR: lymphocyte percentage
W-SCC: absolute value of lymphocyte
RDW-SD: red blood cell volume distribution width-standard deviation
RDW-CV: red blood cell volume distribution width-coefficient of variation
PDW: platelet distribution width
MPV: mean platelet volume
P-LCR: platelet large cell ratio
GLUC: total plasma glucose
UREA: urea nitrogen
TCHE: total plasma cholesterol
TG: triglyceride
NO: nitric oxide
ET: endothelin
Ang II: angiotensin II
RLP: recombinant *Lb. plantarum* NC8
